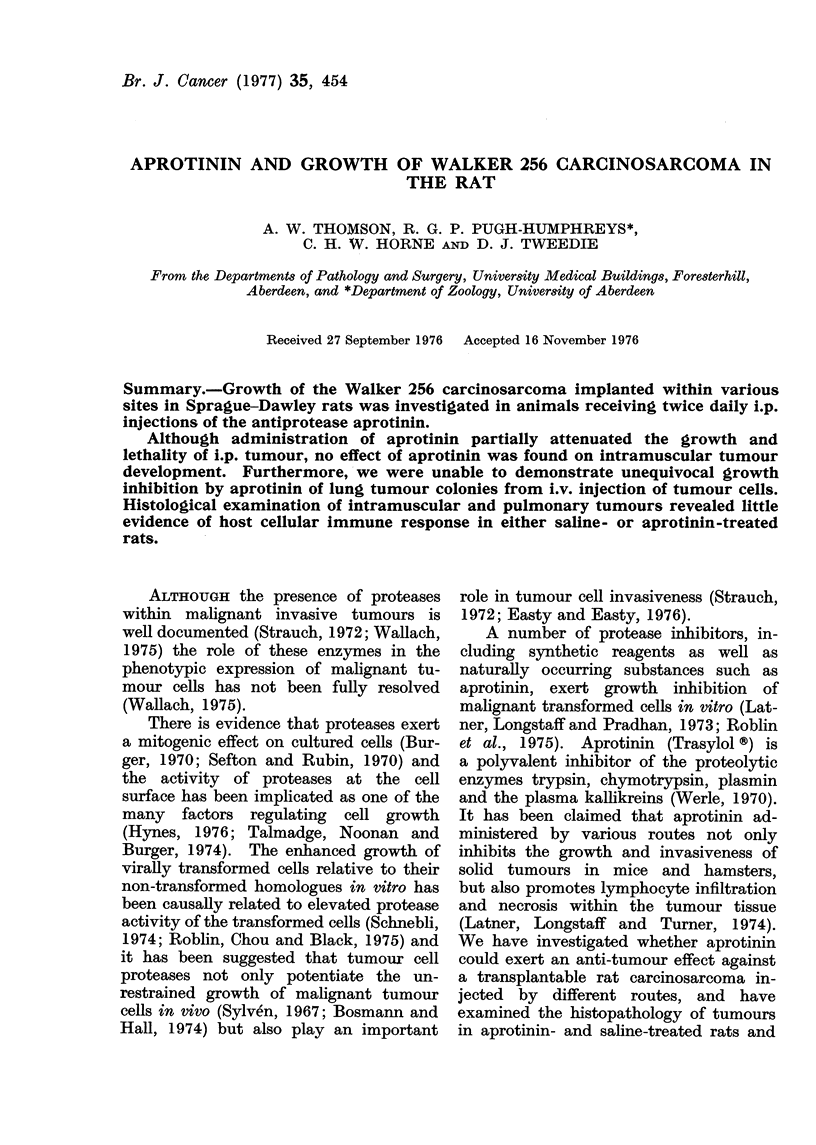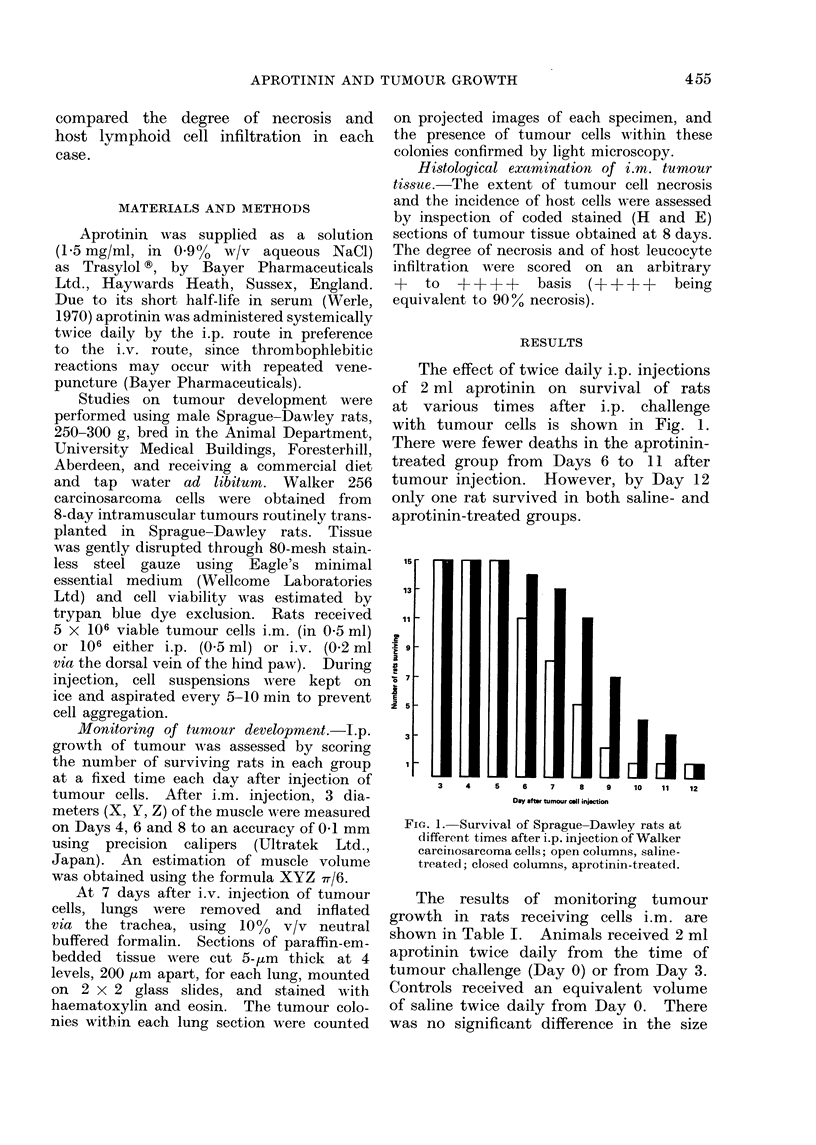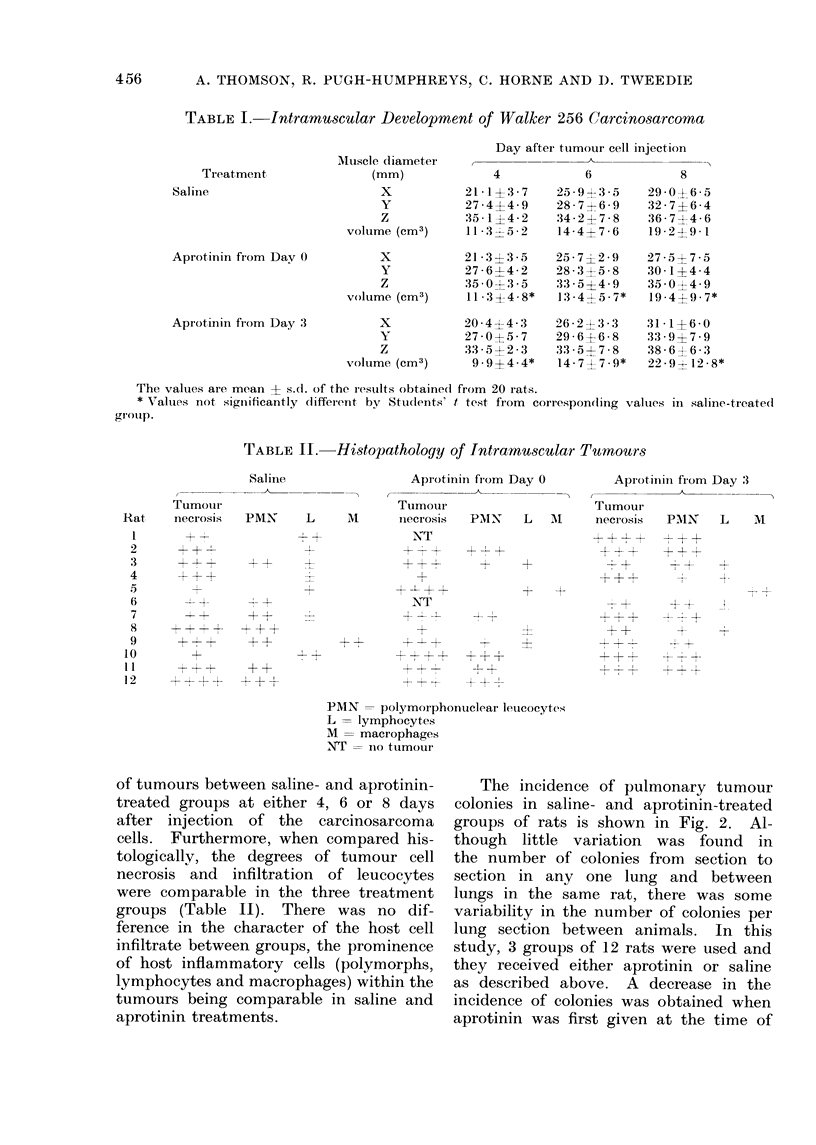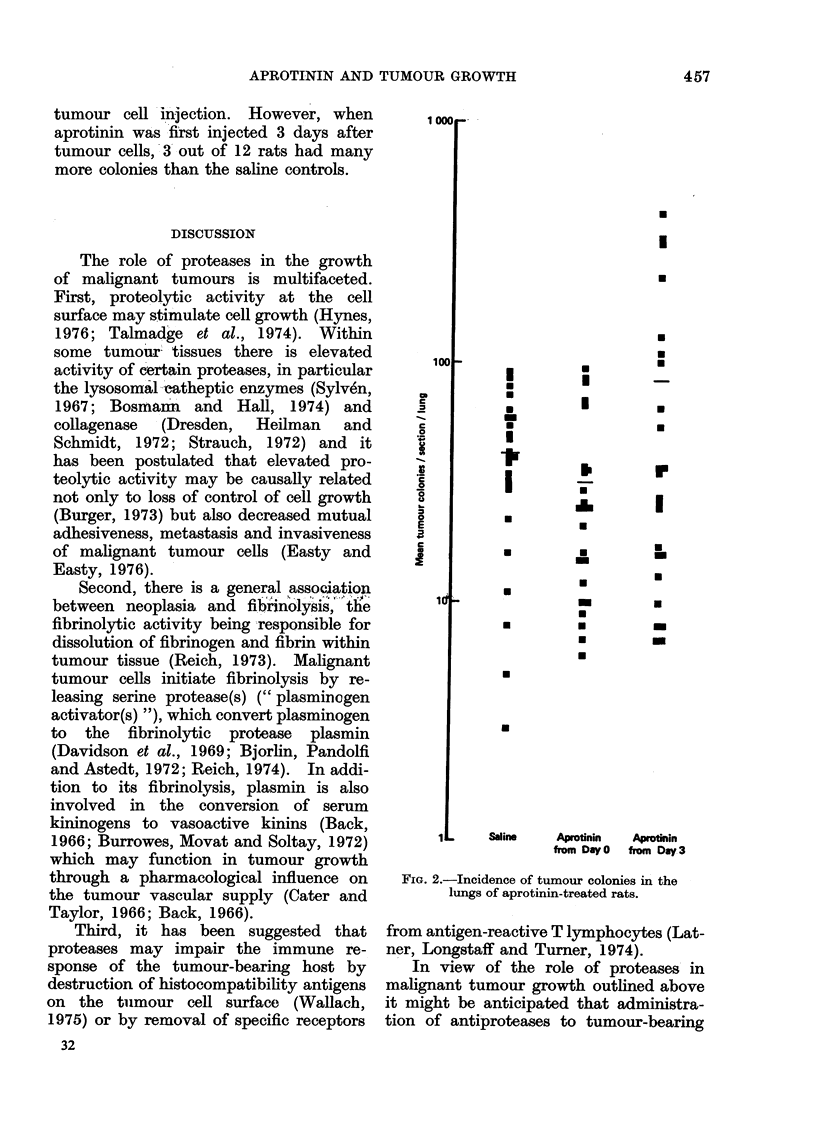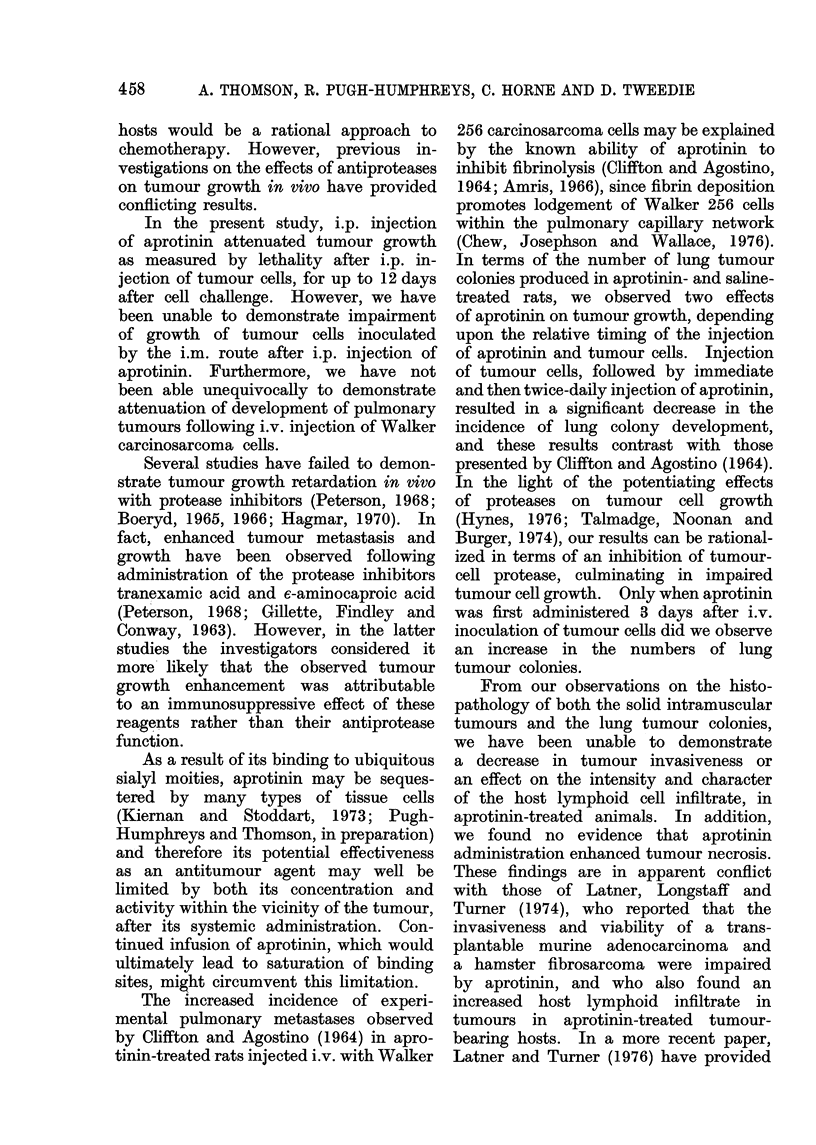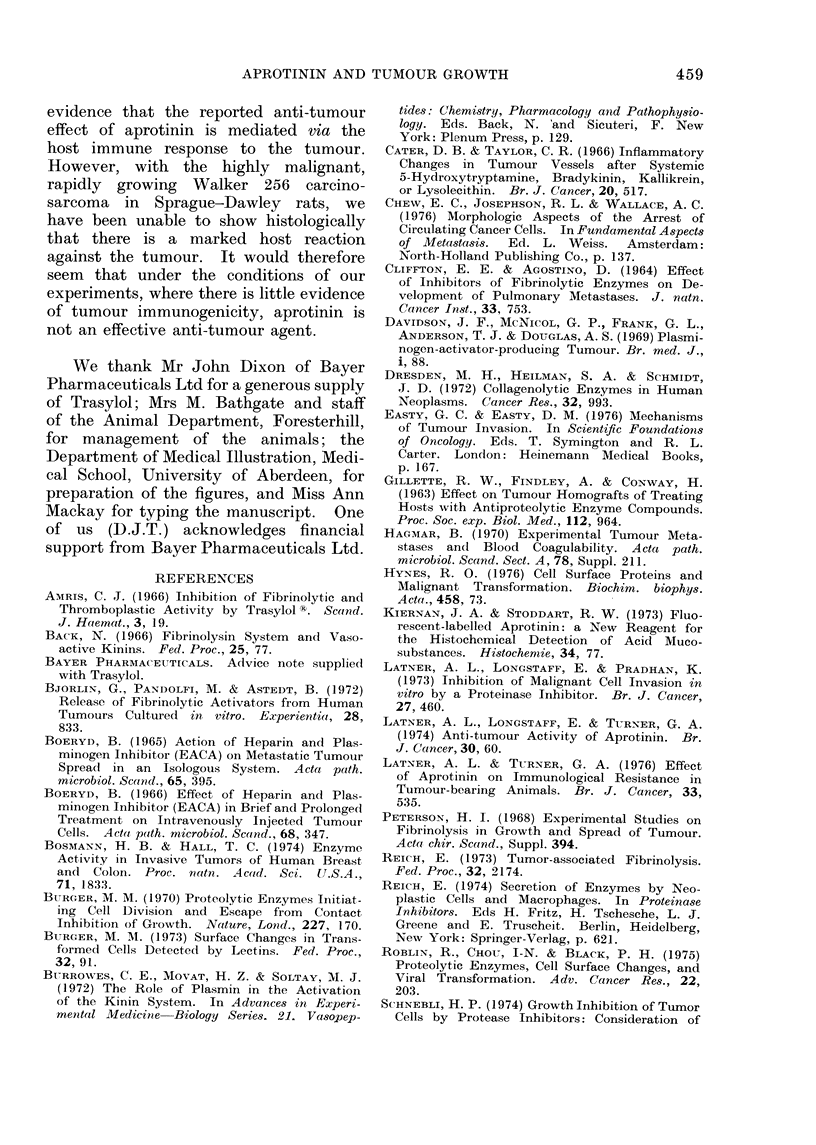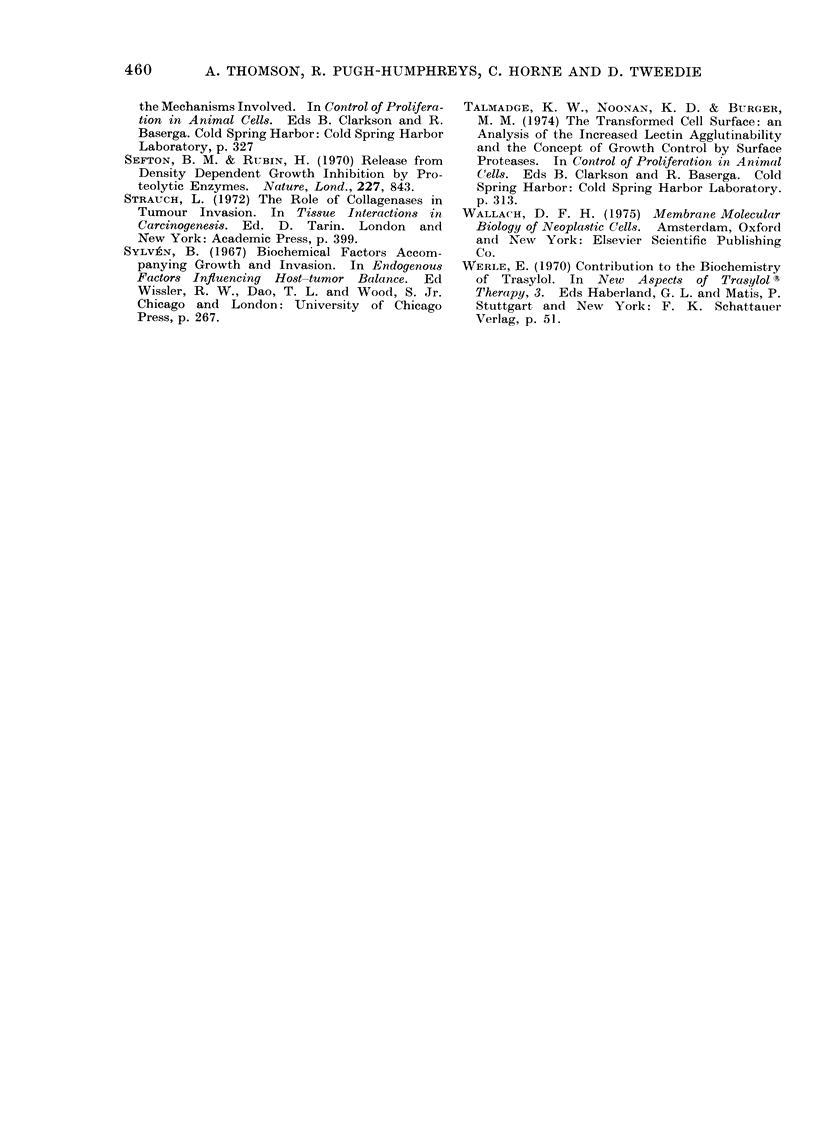# Aprotinin and growth of Walker 256 carcinosarcoma in the rat.

**DOI:** 10.1038/bjc.1977.68

**Published:** 1977-04

**Authors:** A. W. Thomson, R. G. Pugh-Humphreys, C. H. Horne, D. J. Tweedle

## Abstract

Growth of the Walker 256 carcinosarcoma implanted within various sites in Spraque-Dawley rats was investigated in animals receiving twice daily i.p. injections of the antiprotease aprotinin. Although administration of aprotinin partially attenuated the growth and lethality of i.p. tumour, no effect of aprotinin was found on intramuscular tumour development. Furthermore, we were unable to demonstrate unequivocal growth inhibition by aprotinin of lung tumour colonies from i.v. injection of tumour cells. Histological examination of intramuscular and pulmonary tumours revealed little evidence of host cellular immune response in either saline- or aprotinin-treated rats.


Br. J. Cancer (1977) 35, 454

### APROTININ AND GROWTH OF WALKER 256 CARCINOSARCOMA IN THE RAT

A. W. THOMSON, R. G. P. PUGH-HUMPHREYS*, C. H. W. HORNE AND D. J. TWEEDIE

*From the Departments of Pathology and Surgery, University Medical Buildings, Foresterhill, Aberdeen, and *Department of Zoology, University of Aberdeen*

Received 27 September 1976 Accepted 16 November 1976

**Summary.—Growth of the Walker 256 carcinosarcoma implanted within various sites in Sprague–Dawley rats was investigated in animals receiving twice daily i.p. injections of the antiprotease aprotinin.**

**Although administration of aprotinin partially attenuated the growth and lethality of i.p. tumour, no effect of aprotinin was found on intramuscular tumour development. Furthermore, we were unable to demonstrate unequivocal growth inhibition by aprotinin of lung tumour colonies from i.v. injection of tumour cells. Histological examination of intramuscular and pulmonary tumours revealed little evidence of host cellular immune response in either saline- or aprotinin-treated rats.**

ALTHOUGH the presence of proteases within malignant invasive tumours is well documented (Strauch, 1972; Wallach, 1975) the role of these enzymes in the phenotypic expression of malignant tumour cells has not been fully resolved (Wallach, 1975).

There is evidence that proteases exert a mitogenic effect on cultured cells (Burger, 1970; Sefton and Rubin, 1970) and the activity of proteases at the cell surface has been implicated as one of the many factors regulating cell growth (Hynes, 1976; Talmadge, Noonan and Burger, 1974). The enhanced growth of virally transformed cells relative to their non-transformed homologues *in vitro* has been causally related to elevated protease activity of the transformed cells (Schnebli, 1974; Roblin, Chou and Black, 1975) and it has been suggested that tumour cell proteases not only potentiate the unrestrained growth of malignant tumour cells *in vivo* (Sylvén, 1967; Bosmann and Hall, 1974) but also play an important role in tumour cell invasiveness (Strauch, 1972; Easty and Easty, 1976).

A number of protease inhibitors, including synthetic reagents as well as naturally occurring substances such as aprotinin, exert growth inhibition of malignant transformed cells *in vitro* (Latner, Longstaff and Pradhan, 1973; Roblin *et al.*, 1975). Aprotinin (Trasylol ®) is a polyvalent inhibitor of the proteolytic enzymes trypsin, chymotrypsin, plasmin and the plasma kallikreins (Werle, 1970). It has been claimed that aprotinin administered by various routes not only inhibits the growth and invasiveness of solid tumours in mice and hamsters, but also promotes lymphocyte infiltration and necrosis within the tumour tissue (Latner, Longstaff and Turner, 1974). We have investigated whether aprotinin could exert an anti-tumour effect against a transplantable rat carcinosarcoma injected by different routes, and have examined the histopathology of tumours in aprotinin- and saline-treated rats and compared the degree of necrosis and host lymphoid cell infiltration in each case.

### MATERIALS AND METHODS

Aprotinin was supplied as a solution (1·5 mg/ml, in 0·9% w/v aqueous NaCl) as Trasylol®, by Bayer Pharmaceuticals Ltd., Haywards Heath, Sussex, England. Due to its short half-life in serum (Werle, 1970) aprotinin was administered systemically twice daily by the i.p. route in preference to the i.v. route, since thrombophlebitic reactions may occur with repeated venepuncture (Bayer Pharmaceuticals).

Studies on tumour development were performed using male Sprague–Dawley rats, 250–300 g, bred in the Animal Department, University Medical Buildings, Foresterhill, Aberdeen, and receiving a commercial diet and tap water *ad libitum*. Walker 256 carcinosarcoma cells were obtained from 8-day intramuscular tumours routinely transplanted in Sprague–Dawley rats. Tissue was gently disrupted through 80-mesh stainless steel gauze using Eagle's minimal essential medium (Wellcome Laboratories Ltd) and cell viability was estimated by trypan blue dye exclusion. Rats received $5 \times 10^6$ viable tumour cells i.m. (in 0·5 ml) or $10^6$ either i.p. (0·5 ml) or i.v. (0·2 ml *via* the dorsal vein of the hind paw). During injection, cell suspensions were kept on ice and aspirated every 5–10 min to prevent cell aggregation.

*Monitoring of tumour development.*—I.p. growth of tumour was assessed by scoring the number of surviving rats in each group at a fixed time each day after injection of tumour cells. After i.m. injection, 3 diameters (X, Y, Z) of the muscle were measured on Days 4, 6 and 8 to an accuracy of 0·1 mm using precision calipers (Ultratek Ltd., Japan). An estimation of muscle volume was obtained using the formula XYZ $\pi/6$.

At 7 days after i.v. injection of tumour cells, lungs were removed and inflated *via* the trachea, using 10% v/v neutral buffered formalin. Sections of paraffin-embedded tissue were cut 5-$\mu$m thick at 4 levels, 200 $\mu$m apart, for each lung, mounted on 2 × 2 glass slides, and stained with haematoxylin and eosin. The tumour colonies within each lung section were counted on projected images of each specimen, and the presence of tumour cells within these colonies confirmed by light microscopy.

*Histological examination of i.m. tumour tissue.*—The extent of tumour cell necrosis and the incidence of host cells were assessed by inspection of coded stained (H and E) sections of tumour tissue obtained at 8 days. The degree of necrosis and of host leucocyte infiltration were scored on an arbitrary + to ++++ basis (++++ being equivalent to 90% necrosis).

### RESULTS

The effect of twice daily i.p. injections of 2 ml aprotinin on survival of rats at various times after i.p. challenge with tumour cells is shown in Fig. 1. There were fewer deaths in the aprotinin-treated group from Days 6 to 11 after tumour injection. However, by Day 12 only one rat survived in both saline- and aprotinin-treated groups.

FIG. 1.—Survival of Sprague–Dawley rats at different times after i.p. injection of Walker carcinosarcoma cells; open columns, saline-treated; closed columns, aprotinin-treated.

The results of monitoring tumour growth in rats receiving cells i.m. are shown in Table I. Animals received 2 ml aprotinin twice daily from the time of tumour challenge (Day 0) or from Day 3. Controls received an equivalent volume of saline twice daily from Day 0. There was no significant difference in the size

TABLE I.—*Intramuscular Development of Walker 256 Carcinosarcoma*

| Treatment | Muscle diameter (mm) | Day after tumour cell injection 4 | 6 | 8 |
|---|---|---|---|---|
| Saline | X | 21·1±3·7 | 25·9±3·5 | 29·0±6·5 |
| | Y | 27·4±4·9 | 28·7±6·9 | 32·7±6·4 |
| | Z | 35·1±4·2 | 34·2±7·8 | 36·7±4·6 |
| | volume (cm³) | 11·3±5·2 | 14·4±7·6 | 19·2±9·1 |
| Aprotinin from Day 0 | X | 21·3±3·5 | 25·7±2·9 | 27·5±7·5 |
| | Y | 27·6±4·2 | 28·3±5·8 | 30·1±4·4 |
| | Z | 35·0±3·5 | 33·5±4·9 | 35·0±4·9 |
| | volume (cm³) | 11·3±4·8* | 13·4±5·7* | 19·4±9·7* |
| Aprotinin from Day 3 | X | 20·4±4·3 | 26·2±3·3 | 31·1±6·0 |
| | Y | 27·0±5·7 | 29·6±6·8 | 33·9±7·9 |
| | Z | 33·5±2·3 | 33·5±7·8 | 38·6±6·3 |
| | volume (cm³) | 9·9±4·4* | 14·7±7·9* | 22·9±12·8* |

The values are mean ± s.d. of the results obtained from 20 rats.

* Values not significantly different by Students' *t* test from corresponding values in saline-treated group.

TABLE II.—*Histopathology of Intramuscular Tumours*

| | Saline | | | | Aprotinin from Day 0 | | | | Aprotinin from Day 3 | | | |
|---|---|---|---|---|---|---|---|---|---|---|---|---|
| Rat | Tumour necrosis | PMN | L | M | Tumour necrosis | PMN | L | M | Tumour necrosis | PMN | L | M |
| 1 | ++ | | ++ | | NT | | | | ++++ | +++ | | |
| 2 | +++ | | + | | +++ | +++ | | | +++ | +++ | | |
| 3 | +++ | ++ | ± | | +++ | + | + | | ++ | ++ | + | |
| 4 | +++ | | ± | | + | | | | +++ | + | + | |
| 5 | + | | + | | ++++ | | + | + | | | | ++ |
| 6 | ++ | ++ | | | NT | | | | ++ | ++ | ± | |
| 7 | ++ | ++ | ± | | +++ | ++ | | | +++ | +++ | | |
| 8 | ++++ | +++ | | | + | | ± | | ++ | + | + | |
| 9 | +++ | ++ | | ++ | +++ | + | ± | | +++ | ++ | | |
| 10 | + | | ++ | | ++++ | +++ | | | +++ | +++ | | |
| 11 | +++ | ++ | | | +++ | ++ | | | +++ | +++ | | |
| 12 | ++++ | +++ | | | +++ | +++ | | | | | | |

PMN = polymorphonuclear leucocytes

L = lymphocytes

M = macrophages

NT = no tumour of tumours between saline- and aprotinin-treated groups at either 4, 6 or 8 days after injection of the carcinosarcoma cells. Furthermore, when compared histologically, the degrees of tumour cell necrosis and infiltration of leucocytes were comparable in the three treatment groups (Table II). There was no difference in the character of the host cell infiltrate between groups, the prominence of host inflammatory cells (polymorphs, lymphocytes and macrophages) within the tumours being comparable in saline and aprotinin treatments.

The incidence of pulmonary tumour colonies in saline- and aprotinin-treated groups of rats is shown in Fig. 2. Although little variation was found in the number of colonies from section to section in any one lung and between lungs in the same rat, there was some variability in the number of colonies per lung section between animals. In this study, 3 groups of 12 rats were used and they received either aprotinin or saline as described above. A decrease in the incidence of colonies was obtained when aprotinin was first given at the time of tumour cell injection. However, when aprotinin was first injected 3 days after tumour cells, 3 out of 12 rats had many more colonies than the saline controls.

DISCUSSION

The role of proteases in the growth of malignant tumours is multifaceted. First, proteolytic activity at the cell surface may stimulate cell growth (Hynes, 1976; Talmadge *et al.*, 1974). Within some tumour tissues there is elevated activity of certain proteases, in particular the lysosomal catheptic enzymes (Sylvén, 1967; Bosmann and Hall, 1974) and collagenase (Dresden, Heilman and Schmidt, 1972; Strauch, 1972) and it has been postulated that elevated proteolytic activity may be causally related not only to loss of control of cell growth (Burger, 1973) but also decreased mutual adhesiveness, metastasis and invasiveness of malignant tumour cells (Easty and Easty, 1976).

Second, there is a general association between neoplasia and fibrinolysis, the fibrinolytic activity being responsible for dissolution of fibrinogen and fibrin within tumour tissue (Reich, 1973). Malignant tumour cells initiate fibrinolysis by releasing serine protease(s) (" plasminogen activator(s) "), which convert plasminogen to the fibrinolytic protease plasmin (Davidson *et al.*, 1969; Bjorlin, Pandolfi and Astedt, 1972; Reich, 1974). In addition to its fibrinolysis, plasmin is also involved in the conversion of serum kininogens to vasoactive kinins (Back, 1966; Burrowes, Movat and Soltay, 1972) which may function in tumour growth through a pharmacological influence on the tumour vascular supply (Cater and Taylor, 1966; Back, 1966).

Third, it has been suggested that proteases may impair the immune response of the tumour-bearing host by destruction of histocompatibility antigens on the tumour cell surface (Wallach, 1975) or by removal of specific receptors

FIG. 2.—Incidence of tumour colonies in the lungs of aprotinin-treated rats.

from antigen-reactive T lymphocytes (Latner, Longstaff and Turner, 1974).

In view of the role of proteases in malignant tumour growth outlined above it might be anticipated that administration of antiproteases to tumour-bearing hosts would be a rational approach to chemotherapy. However, previous investigations on the effects of antiproteases on tumour growth *in vivo* have provided conflicting results.

In the present study, i.p. injection of aprotinin attenuated tumour growth as measured by lethality after i.p. injection of tumour cells, for up to 12 days after cell challenge. However, we have been unable to demonstrate impairment of growth of tumour cells inoculated by the i.m. route after i.p. injection of aprotinin. Furthermore, we have not been able unequivocally to demonstrate attenuation of development of pulmonary tumours following i.v. injection of Walker carcinosarcoma cells.

Several studies have failed to demonstrate tumour growth retardation *in vivo* with protease inhibitors (Peterson, 1968; Boeryd, 1965, 1966; Hagmar, 1970). In fact, enhanced tumour metastasis and growth have been observed following administration of the protease inhibitors tranexamic acid and $\epsilon$-aminocaproic acid (Peterson, 1968; Gillette, Findley and Conway, 1963). However, in the latter studies the investigators considered it more likely that the observed tumour growth enhancement was attributable to an immunosuppressive effect of these reagents rather than their antiprotease function.

As a result of its binding to ubiquitous sialyl moities, aprotinin may be sequestered by many types of tissue cells (Kiernan and Stoddart, 1973; Pugh-Humphreys and Thomson, in preparation) and therefore its potential effectiveness as an antitumour agent may well be limited by both its concentration and activity within the vicinity of the tumour, after its systemic administration. Continued infusion of aprotinin, which would ultimately lead to saturation of binding sites, might circumvent this limitation.

The increased incidence of experimental pulmonary metastases observed by Cliffton and Agostino (1964) in aprotinin-treated rats injected i.v. with Walker 256 carcinosarcoma cells may be explained by the known ability of aprotinin to inhibit fibrinolysis (Cliffton and Agostino, 1964; Amris, 1966), since fibrin deposition promotes lodgement of Walker 256 cells within the pulmonary capillary network (Chew, Josephson and Wallace, 1976). In terms of the number of lung tumour colonies produced in aprotinin- and saline-treated rats, we observed two effects of aprotinin on tumour growth, depending upon the relative timing of the injection of aprotinin and tumour cells. Injection of tumour cells, followed by immediate and then twice-daily injection of aprotinin, resulted in a significant decrease in the incidence of lung colony development, and these results contrast with those presented by Cliffton and Agostino (1964). In the light of the potentiating effects of proteases on tumour cell growth (Hynes, 1976; Talmadge, Noonan and Burger, 1974), our results can be rationalized in terms of an inhibition of tumour-cell protease, culminating in impaired tumour cell growth. Only when aprotinin was first administered 3 days after i.v. inoculation of tumour cells did we observe an increase in the numbers of lung tumour colonies.

From our observations on the histopathology of both the solid intramuscular tumours and the lung tumour colonies, we have been unable to demonstrate a decrease in tumour invasiveness or an effect on the intensity and character of the host lymphoid cell infiltrate, in aprotinin-treated animals. In addition, we found no evidence that aprotinin administration enhanced tumour necrosis. These findings are in apparent conflict with those of Latner, Longstaff and Turner (1974), who reported that the invasiveness and viability of a transplantable murine adenocarcinoma and a hamster fibrosarcoma were impaired by aprotinin, and who also found an increased host lymphoid infiltrate in tumours in aprotinin-treated tumour-bearing hosts. In a more recent paper, Latner and Turner (1976) have provided evidence that the reported anti-tumour effect of aprotinin is mediated *via* the host immune response to the tumour. However, with the highly malignant, rapidly growing Walker 256 carcinosarcoma in Sprague–Dawley rats, we have been unable to show histologically that there is a marked host reaction against the tumour. It would therefore seem that under the conditions of our experiments, where there is little evidence of tumour immunogenicity, aprotinin is not an effective anti-tumour agent.

We thank Mr John Dixon of Bayer Pharmaceuticals Ltd for a generous supply of Trasylol; Mrs M. Bathgate and staff of the Animal Department, Foresterhill, for management of the animals; the Department of Medical Illustration, Medical School, University of Aberdeen, for preparation of the figures, and Miss Ann Mackay for typing the manuscript. One of us (D.J.T.) acknowledges financial support from Bayer Pharmaceuticals Ltd.

### REFERENCES

AMRIS, C. J. (1966) Inhibition of Fibrinolytic and Thromboplastic Activity by Trasylol®. *Scand. J. Haemat.*, **3**, 19.

BACK, N. (1966) Fibrinolysin System and Vasoactive Kinins. *Fed. Proc.*, **25**, 77.

BAYER PHARMACEUTICALS. Advice note supplied with Trasylol.

BJORLIN, G., PANDOLFI, M. & ASTEDT, B. (1972) Release of Fibrinolytic Activators from Human Tumours Cultured *in vitro*. *Experientia*, **28**, 833.

BOERYD, B. (1965) Action of Heparin and Plasminogen Inhibitor (EACA) on Metastatic Tumour Spread in an Isologous System. *Acta path. microbiol. Scand.*, **65**, 395.

BOERYD, B. (1966) Effect of Heparin and Plasminogen Inhibitor (EACA) in Brief and Prolonged Treatment on Intravenously Injected Tumour Cells. *Acta path. microbiol. Scand.*, **68**, 347.

BOSMANN, H. B. & HALL, T. C. (1974) Enzyme Activity in Invasive Tumors of Human Breast and Colon. *Proc. natn. Acad. Sci. U.S.A.*, **71**, 1833.

BURGER, M. M. (1970) Proteolytic Enzymes Initiating Cell Division and Escape from Contact Inhibition of Growth. *Nature, Lond.*, **227**, 170.

BURGER, M. M. (1973) Surface Changes in Transformed Cells Detected by Lectins. *Fed. Proc.*, **32**, 91.

BURROWES, C. E., MOVAT, H. Z. & SOLTAY, M. J. (1972) The Role of Plasmin in the Activation of the Kinin System. In *Advances in Experimental Medicine—Biology Series. 21. Vasopeptides: Chemistry, Pharmacology and Pathophysiology.* Eds. Back, N. 'and Sicuteri, F. New York: Plenum Press, p. 129.

CATER, D. B. & TAYLOR, C. R. (1966) Inflammatory Changes in Tumour Vessels after Systemic 5-Hydroxytryptamine, Bradykinin, Kallikrein, or Lysolecithin. *Br. J. Cancer*, **20**, 517.

CHEW, E. C., JOSEPHSON, R. L. & WALLACE, A. C. (1976) Morphologic Aspects of the Arrest of Circulating Cancer Cells. In *Fundamental Aspects of Metastasis.* Ed. L. Weiss. Amsterdam: North-Holland Publishing Co., p. 137.

CLIFFTON, E. E. & AGOSTINO, D. (1964) Effect of Inhibitors of Fibrinolytic Enzymes on Development of Pulmonary Metastases. *J. natn. Cancer Inst.*, **33**, 753.

DAVIDSON, J. F., MCNICOL, G. P., FRANK, G. L., ANDERSON, T. J. & DOUGLAS, A. S. (1969) Plasminogen-activator-producing Tumour. *Br. med. J.*, **i**, 88.

DRESDEN, M. H., HEILMAN, S. A. & SCHMIDT, J. D. (1972) Collagenolytic Enzymes in Human Neoplasms. *Cancer Res.*, **32**, 993.

EASTY, G. C. & EASTY, D. M. (1976) Mechanisms of Tumour Invasion. In *Scientific Foundations of Oncology.* Eds. T. Symington and R. L. Carter. London: Heinemann Medical Books, p. 167.

GILLETTE, R. W., FINDLEY, A. & CONWAY, H. (1963) Effect on Tumour Homografts of Treating Hosts with Antiproteolytic Enzyme Compounds. *Proc. Soc. exp. Biol. Med.*, **112**, 964.

HAGMAR, B. (1970) Experimental Tumour Metastases and Blood Coagulability. *Acta path. microbiol. Scand. Sect. A*, **78**, Suppl. 211.

HYNES, R. O. (1976) Cell Surface Proteins and Malignant Transformation. *Biochim. biophys. Acta.*, **458**, 73.

KIERNAN, J. A. & STODDART, R. W. (1973) Fluorescent-labelled Aprotinin: a New Reagent for the Histochemical Detection of Acid Mucosubstances. *Histochemie*, **34**, 77.

LATNER, A. L., LONGSTAFF, E. & PRADHAN, K. (1973) Inhibition of Malignant Cell Invasion *in vitro* by a Proteinase Inhibitor. *Br. J. Cancer*, **27**, 460.

LATNER, A. L., LONGSTAFF, E. & TURNER, G. A. (1974) Anti-tumour Activity of Aprotinin. *Br. J. Cancer*, **30**, 60.

LATNER, A. L. & TURNER, G. A. (1976) Effect of Aprotinin on Immunological Resistance in Tumour-bearing Animals. *Br. J. Cancer*, **33**, 535.

PETERSON, H. I. (1968) Experimental Studies on Fibrinolysis in Growth and Spread of Tumour. *Acta chir. Scand.*, Suppl. **394**.

REICH, E. (1973) Tumor-associated Fibrinolysis. *Fed. Proc.*, **32**, 2174.

REICH, E. (1974) Secretion of Enzymes by Neoplastic Cells and Macrophages. In *Proteinase Inhibitors.* Eds H. Fritz, H. Tschesche, L. J. Greene and E. Truscheit. Berlin, Heidelberg, New York: Springer-Verlag, p. 621.

ROBLIN, R., CHOU, I-N. & BLACK, P. H. (1975) Proteolytic Enzymes, Cell Surface Changes, and Viral Transformation. *Adv. Cancer Res.*, **22**, 203.

SCHNEBLI, H. P. (1974) Growth Inhibition of Tumor Cells by Protease Inhibitors: Consideration of the Mechanisms Involved. In *Control of Proliferation in Animal Cells.* Eds B. Clarkson and R. Baserga. Cold Spring Harbor: Cold Spring Harbor Laboratory, p. 327

SEFTON, B. M. & RUBIN, H. (1970) Release from Density Dependent Growth Inhibition by Proteolytic Enzymes. *Nature, Lond.*, **227**, 843.

STRAUCH, L. (1972) The Role of Collagenases in Tumour Invasion. In *Tissue Interactions in Carcinogenesis.* Ed. D. Tarin. London and New York: Academic Press, p. 399.

SYLVÉN, B. (1967) Biochemical Factors Accompanying Growth and Invasion. In *Endogenous Factors Influencing Host-tumor Balance.* Ed Wissler, R. W., Dao, T. L. and Wood, S. Jr. Chicago and London: University of Chicago Press, p. 267.

TALMADGE, K. W., NOONAN, K. D. & BURGER, M. M. (1974) The Transformed Cell Surface: an Analysis of the Increased Lectin Agglutinability and the Concept of Growth Control by Surface Proteases. In *Control of Proliferation in Animal Cells.* Eds B. Clarkson and R. Baserga. Cold Spring Harbor: Cold Spring Harbor Laboratory. p. 313.

WALLACH, D. F. H. (1975) *Membrane Molecular Biology of Neoplastic Cells.* Amsterdam, Oxford and New York: Elsevier Scientific Publishing Co.

WERLE, E. (1970) Contribution to the Biochemistry of Trasylol. In *New Aspects of Trasylol® Therapy, 3.* Eds Haberland, G. L. and Matis, P. Stuttgart and New York: F. K. Schattauer Verlag, p. 51.